# Neuroanatomical and Neuropsychological Markers of Amnestic MCI: A Three-Year Longitudinal Study in Individuals Unaware of Cognitive Decline

**DOI:** 10.3389/fnagi.2017.00034

**Published:** 2017-02-22

**Authors:** Katharina S. Goerlich, Mikhail Votinov, Ellen Dicks, Sinika Ellendt, Gábor Csukly, Ute Habel

**Affiliations:** ^1^Department of Psychiatry, Psychotherapy and Psychosomatics, Medical Faculty, RWTH Aachen UniversityAachen, Germany; ^2^Jülich Aachen Research Alliance (JARA) – Translational Brain MedicineAachen, Germany; ^3^Institute of Neuroscience and Medicine (INM-10), Research Centre JülichJülich, Germany; ^4^Alzheimer Center and Department of Neurology, Neuroscience Campus Amsterdam, VU University Medical CenterAmsterdam, Netherlands; ^5^Department of Psychiatry and Psychotherapy, Semmelweis UniversityBudapest, Hungary

**Keywords:** mild cognitive impairment, voxel-based morphometry, subjective cognitive complaints, gray matter atrophy, amygdala, hippocampus, longitudinal

## Abstract

Structural brain changes underlying mild cognitive impairment (MCI) have been well-researched, but most previous studies required subjective cognitive complaints (SCC) as a diagnostic criterion, diagnosed MCI based on a single screening test or lacked analyses in relation to neuropsychological impairment. This longitudinal voxel-based morphometry study aimed to overcome these limitations: The relationship between regional gray matter (GM) atrophy and behavioral performance was investigated over the course of 3 years in individuals unaware of cognitive decline, identified as amnestic MCI based on an extensive neuropsychological test battery. Region of interest analyses revealed GM atrophy in the left amygdala, hippocampus, and parahippocampus in MCI individuals compared to normally aging participants, which was specifically related to verbal memory impairment and evident already at the first measurement point. These findings demonstrate that GM atrophy is detectable in individuals with amnestic MCI despite unawareness of beginning cognitive decline. Thus, individuals with GM atrophy in regions associated with verbal memory impairment do not necessarily need to experience SCC before meeting neuropsychological criteria for MCI. These results have important implications for future research and diagnostic procedures of MCI.

## Introduction

Age-related neurodegenerative diseases such as Alzheimer’s disease (AD) impose a high social and financial burden for society that will increase in the following decades, given predictions of a 9% increase of people above 60% in 2050 (United Nations, Department of Economic and Social Affairs, 2013). Along with population aging, the prevalence of AD (currently 4.7%) is predicted to increase by approximately 225% by 2050 (Prince et al., 2013).

Memory disturbances constitute early symptoms of AD, and with progressing impairment, other domains become affected, including language, problem-solving, and visuospatial perception (for a review, see Dubois et al., 2016). The neuropathological hallmarks of AD are brain atrophy, extracellular amyloid plaques, and neurofibrillary tangles (Kandel et al., 2013), yet its underlying cause remains unidentified (Zetterberg and Mattsson, 2014). Early diagnosis is in dire need for disease prevention and the development of new treatment strategies. Detecting AD at an early time point would enable early intervention and a timely start of treatment, possibly preventing disease progression.

A promising endeavor to provide such early diagnosis lies in the identification of the transitional state between normal aging and pathological cognitive decline. Since 1997, the term mild cognitive impairment (MCI) is used to mark the difference between continuous levels of cognitive impairment in normally aging controls compared to AD patients (Petersen et al., 1997). The concept of MCI is now widely accepted and continues to receive great attention in the literature as it represents a possible treatment target for AD. Its predictive power is reflected in annual conversion rates of up to 15% from MCI to AD (DeCarli, 2003) compared to conversion rates of 1–2% from normal aging to AD (Petersen et al., 1999).

Currently, MCI is diagnosed by: (a) abnormal cognitive function adjusted for age and education level, (b) self-reported cognitive complaints, (c) normal activities of daily living, and (d) absence of a dementia diagnosis (Winblad et al., 2004). Impairment severity is commonly assessed on the basis of general cognitive screening tests, such as the Mini-Mental State Examination (MMSE). However, such tests have been criticized for being not sufficiently specific regarding the subtle nature of MCI (Nickl-Jockschat et al., 2012; Forlenza et al., 2013). Even if specific neuropsychological tests for MCI diagnosis are used, studies often rely on only one diagnostic test (Bondi et al., 2014). Moreover, there is great variation in how the criteria for aberrant cognitive function are implemented. Thus, there is no standardized approach for diagnosing MCI, and prevalence rates are therefore highly dependent on the classification scheme used (Chapman et al., 2016; Clark et al., 2016). Furthermore, MCI was originally conceptualized as a prodromal stage for AD and thus focused on memory impairment (Petersen et al., 1999). However, MCI has been revealed to be a much broader construct affecting several cognitive domains (Winblad et al., 2004). Thus, extensive neuropsychological testing is required to identify early-stage MCI.

Critically, doubts have arisen regarding subjective cognitive complaints (SCC), which are currently implemented in the diagnosis of MCI. While these may be a marker of MCI (Clark et al., 2016), including SCC may lead to false omission of possible MCI candidates who are misdiagnosed as healthily aging individuals because they are not yet aware of beginning cognitive impairment (Edmonds et al., 2014). Such a lack of awareness has indeed been identified by several studies (e.g., Purser et al., 2006; Lenehan et al., 2012; Fragkiadaki et al., 2016; for a review, see Roberts et al., 2009), and the inclusion of SCC resulted in increased rates of false negative and false positive diagnoses (Lenehan et al., 2012). Moreover, subjective complaints are strongly related to individual differences in depression and neuroticism, casting further doubt on their reliability as a diagnostic marker of MCI (Reid and MacLullich, 2006). Taken together, the drawbacks of including SCC in the diagnosis of MCI seem to outweigh its benefits. Thus, the present study took a novel approach by investigating MCI in elderly individuals unaware of any cognitive impairment.

In addition to more thorough neuropsychological testing, the need for neuroanatomical biomarkers of MCI has been recognized (Ruan et al., 2016). For AD, the most widely studied biomarkers are: decreased cerebrospinal fluid amyloid beta (CSF Aβ), increased CSF tau, decreased fluorodeoxyglucose uptake on positron-emission tomography (FDG-PET), PET amyloid imaging, and structural MRI measures of cerebral atrophy. Importantly, strong evidence suggests that MRI, FDG-PET, and CSF tau biomarkers are already abnormal in the MCI phase of AD (Ewers et al., 2007; Sluimer et al., 2008; Shaw et al., 2009), and while both CSF tau and MRI are predictive of conversion from MCI to AD, the predictive power of structural MRI is greater. Moreover, cognitive measures correlate strongly with structural MRI, but not with CSF tau in patients with MCI (Vemuri et al., 2009). Structural MRI studies using longitudinal voxel-based morphometry (VBM) identified the medial temporal lobe (MTL) as a core region for progression of MCI to AD (Ferreira et al., 2011; Nickl-Jockschat et al., 2012; Yang et al., 2012). This is not surprising given that the MTL contains essential structures for memory consolidation (Kandel et al., 2013). The identification of progressing MCI patients can be made apparent already 4 years prior to conversion to AD on the basis of hippocampal atrophy patterns (Adaszewski et al., 2013). Comparing rates of hippocampal atrophy between healthy controls, MCI individuals and AD patients, annual atrophy rates of 1.9–2.8% were observed for the controls, 2.6–3.7% for MCI individuals, and 3.5% for AD patients (Jack et al., 2000). Moreover, meta-analyses indicate that a consistent atrophy pattern underlies MCI, comprising the amygdala, hippocampus, precuneus, and posterior cingulate gyrus (Nickl-Jockschat et al., 2012; Yang et al., 2012).

Although gray matter (GM) changes accompanying MCI have been well-researched, most of the previous studies identified MCI on the basis of general cognitive screening tests such as the MMSE, which may be insufficient to capture the subtle nature of MCI and presumably identifies MCI at a rather late stage, missing the beginning of cognitive decline. Even if the MMSE was not used for MCI diagnosis, previous VBM studies correlated GM atrophy with cognitive decline based on the MMSE and thus lack analyses in relation to specific behavioral impairment (e.g., Jack et al., 2000; Whitwell et al., 2007; Sluimer et al., 2008; Weiner et al., 2013).

The present study aimed to overcome these limitations by taking a novel, more sensitive approach: Elderly volunteers (minimum age: 50 years) from a community sample were included who had no subjective complaints, i.e., were not aware of potential cognitive impairment. MCI was then identified on the basis of a thorough neuropsychological test battery assessing memory, intelligence, executive functions, psychomotor speed, visuo-construction and visuo-spatial skills, attention, and language. To our knowledge, this is the first VBM study investigating the longitudinal trajectory of morphological changes underlying early-stage MCI in individuals still unaware of beginning cognitive impairment. We predicted more pronounced region of interest (ROI) GM atrophy in individuals classified as MCI compared to HCs even without subjective awareness of cognitive deficits, and hypothesized faster atrophy rates in the MCI compared to the HC group. Moreover, we hypothesized ROI GM atrophy to be related to specific behavioral impairment in the MCI group only.

## Materials and Methods

### Participants

Participants for the Helmholtz Alliance for Mental Health in an Aging Society (HelMA) study (Drexler et al., 2013; Chechko et al., 2014; Ellendt et al., 2016) were recruited through visitations to social facilities for elderly people (charity organizations and citizen centers) and advertisements in local newspapers. From 81 volunteers initially participating in the first measurement time point (T_1_), 43 participants (27 women, aged 50–83 years) eventually completed all three measurement time points (T_1_, T_2_, and T_3_) with a mean follow-up interval of 1.12 years, *SD* 0.38 years. Reasons for dropping out varied from refusing to further participate for time reasons or a lack of interest (13 participants), moving to another city (four participants), newly acquired MR contraindications following surgery during the course of the study (six participants), to illness (11 participants) and death (four participants).

Participants were included if they were 50 years or older, had sufficient German language and adequate visual performance abilities. Exclusion criteria comprised a diagnosis of dementia, neurological or psychiatric disorders according to DSM-IV axis I as assessed by the German version of Structured Clinical Interview (SKID; Wittchen et al., 1997), physical disease that could interfere with cognitive performance, lifetime or current drug addiction, seriously reduced vision, inability to follow the protocol, and medication use with possible cognitive side effects. The study was approved by the ethics committee of the medical faculty, RWTH Aachen University. All participants gave written informed consent and were paid for participation.

### Neuropsychological Data Analysis

In addition to several dementia screening tests applied with the objective of eliminating dementia rather than diagnosing MCI, all participants underwent an extensive neuropsychological test battery evaluating memory, intelligence, executive functions, psychomotor speed, visuo-construction and visuo-spatial skills, attention, and language at each time point. **Table 1** provides an overview of all neuropsychological tests used for MCI assessment and diagnosis (for further details, see Drexler et al., 2013; Ellendt et al., 2016). The testing procedure was accomplished by trained psychologists. To counteract learning effects, tests were presented in counterbalanced order. Sessions took place in the mornings and lasted approximately 3 h. Short breaks were offered and if necessary, a second appointment was arranged.

**Table 1:** Neuropsychological test battery used for the assessment and diagnosis of mild cognitive impairment (MCI).

Domain	Test
Memory	Benton Visual Retention Test (BRVT) Wechsler Memory Scale – digit span (WMS-R) California Verbal Learning Test, German version (VLMT)
Intelligence	Premorbid intelligence; Multiple-choice word test, German version (MWT-B)
Executive functions	Cognitive flexibility, divided attention, psychomotor speed: Trail-Making-Test A and B (TMT A/B) Planning and problem-solving: Tower of London (TL-D) Lexical and semantic fluency: Regensburger Wortflüssigkeitstest (RWT)
Dementia screening	Mini-Mental-State-Examination, German version (MMSE) Syndrom Kurz Test (SKT) Test zur Früherkennung von Demenzen (TFDD) Consortium to Establish a Registry for Alzheimer’s Disease (CERAD-PLUS) including language, object naming abilities (Boston Naming Test), verbal learning, visuospatial functions, delayed recall, memory consolidation, recognition, executive functions


The classification criteria for MCI were based on those described by Winblad et al. (2004). That is, (1) greater memory impairment than expected for age, (2) preserved activities of the daily living, and (3) absence of dementia. The criterion of memory impairment greater than expected for age was identified by an impaired score of at least 1.5 standard deviations (SD) below the mean according to normative datasets in at least one test assessing memory functioning (i.e., VLMT, Benton, WMS, memory assessing subtests of the CERAD-Plus battery). If the neuropsychological test comprised multiple subtests (as in VLMT and CERAD), at least two subtests had to indicate impairment, i.e., 1.5 *SD* below normative data, which is considered a conservative method for identifying MCI (Jak et al., 2009). The decision to classify subjects solely on their performance in tests regarding memory functioning can be accounted on the fact that amnestic MCI patients show a higher prevalence to develop AD compared to non-amnestic MCI patients, who have an increased risk of developing other types of dementia (Petersen and Morris, 2005). Note that impairment in cognitive domains other than memory was not observed in this sample. Thus, all MCI participants identified here can be classified as belonging to the single-domain amnestic MCI subtype. Correspondingly, the present results are of relevance specifically to amnestic MCI.

The original classification criteria proposed by Winblad et al. (2004) also include SCC but because the requirement of SCCs could lead to omitting subjects which would otherwise be classified as MCI (Edmonds et al., 2014), this criterion was not applied. In fact, all participants reported not to have experienced any difficulties regarding memory or any other cognitive domain beyond expected for their respective age. Thus, none of the participants were aware of any cognitive impairment.

The neuropsychological data were analyzed in SPSS 20 by means of multivariate analyses of covariance (MANCOVA) comparing performance between the MCI group and the HC group at each time point, corrected for age, gender, and education. The initial significance threshold was *p* < 0.05, and all results were Bonferroni corrected for multiple comparisons.

### VBM Data Analysis

T1 anatomical images (TE: 3.03; TR: 2300 ms; FOV = 256 mm × 256 mm; number of sagittal slices = 176; voxel size: 1 mm × 1 mm × 1 mm) from the 43 study participants were acquired on a 3 Tesla Siemens^®^ Trio MR scanner. Differences in GM volume between the MCI and HC group were assessed using longitudinal VBM (Ashburner and Friston, 2000; Ashburner and Ridgway, 2013) implemented in SPM12 (Wellcome Trust Centre for Neuroimaging, University College London, London, UK). The following preprocessing steps were applied: First, serial longitudinal registration, which produces one midpoint file and Jacobian determinants for each subject at each of the three time points. Next, each subject’s midpoint average was segmented into GM, white matter (WM), and CSF. Then, GM images were computed for each subject at each time point using the respective Jacobian determinants. These images were spatially normalized by creating a customized group-specific template using the DARTEL approach and warping each of the individual GM segmentations onto this template. The warped GM segmentations were modulated to reflect the volume and smoothed using a Gaussian kernel of 8 mm at full width at half-maximum (FWHM). Data quality was ensured by visually checking each T1 image for abnormalities before preprocessing, checking volume orientation before smoothing during preprocessing, and by means of a sample homogeneity check after preprocessing, revealing no outliers.

Region of interest analyses were performed on GM volumes of eight anatomically defined *a priori* ROIs based on MCI meta-analyses (Nickl-Jockschat et al., 2012; Weiner et al., 2013): Left and right amygdala, hippocampus, parahippocampus, and precuneus. Anatomical ROIs for these regions were created using the automatic anatomic labeling (AAL) atlas templates (Tzourio-Mazoyer et al., 2002) provided by the WFU Pickatlas toolbox (Wake Forest School of Medicine, Winston Salem, NC, USA). Mean parameter estimates from these ROIs were extracted using MarsBaR^1^ and analyzed by means of a Linear Mixed Model (LMM) Analysis in SAS 9.2. The factor group (MCI versus HC) was included in the LMM as a between-subjects fixed effect, and the model controlled for age, gender, education, and mean total intracranial volume (TIV, i.e., GM + WM + CSF). An unstructured covariance matrix was used, which was estimated by means of the null model estimation method.

Relationships between mean neuropsychological performance and mean GM volumes of the eight ROIs across the whole sample and within the HC and the MCI group were examined by means of partial correlations controlling for age, gender, education, and TIV. To this end, the residuals of each neuropsychological test as well as the residuals of the GM volumes of each ROI at each measurement point were calculated, controlling for age, gender, education, and TIV, and partial correlations were then performed between the neuropsychological test residuals and the ROI GM volume residuals. The Holm–Bonferroni correction was applied to control for multiple comparisons, resulting in an initial significance threshold of *p* < 0.006 (*p* = 0.05/*n* = 8 ROIs). Moreover, whole-brain analyses comparing GM volumes between the MCI and HC group at each time point were conducted at an uncorrected threshold *p* < 0.001.

## Results

### Neuropsychological Performance

Based on the neuropsychological test battery, 16 participants were classified as MCI (6 male, mean age 66.13, *SD* 8.46 years, mean years of education 9.43, *SD* 1.59), and 27 participants were classified as HC (10 male, mean age 66.15, *SD* 6.15 years, mean years of education 10.5, *SD* 1.88). Groups did not differ in age (*p* > 0.05), but the HC group had more years of education (*p* < 0.05). The results on the neuropsychological tests for the HC and the MCI group are presented in **Table 2**, revealing significantly worse performance of the MCI group compared to the HC group on the total immediate recall and delayed recall subtests of the VLMT and the CERAD. At a lower significance threshold (*p* < 0.05 uncorrected), worse performance was additionally observed in the Benton test (correct drawings and number of mistakes) and the WMS-R subtests digit span forward and backward. The MMSE could not distinguish between the MCI and the HC group, as opposed to the TFDD, which indicated lower performance in MCI compared to HC participants (see **Table 2**). Note that none of the study participants converted to dementia during the course of the investigation.

**Table 2:** Neuropsychological test results for the MCI group compared to the HC group.

	MCI			HC			MCI vs. HC
			
	T_1-3_Mean ± SEM			T_1-3_ Mean ± SEM			T_1-3_Mean
			
	T_1_	T_2_	T_3_	T_1_	T_2_	T_3_	*p*-value
**VLMT**
Total immediate recall		45.56 ± 7.05			54.85 ± 5.75		0.0002**
	43.69	45.75	47.25	52.89	54.30	57.37	
Delayed recall		8.83 ± 2.06			11.82 ± 1.99		0.0001**
	8.56	8.63	9.31	11.15	11.67	12.63	
Lost words		2.98 ± 1.79			1.59 ± 1.33		0.09
	2.75	3.06	3.13	1.85	1.63	1.30	
Correct recognition		13.31 ± 0.92			14.37 ± 0.7		0.01*
	13.5	12.81	13.63	14.11	14.48	14.52	
Recognition residuals		10.63 ± 2.15			12.96 ± 1.63		0.01*
	11.5	9.5	10.88	12.85	12.70	13.33	
**WMS-R**
Digit span forward		6.94 ± 1.64			7.89 ± 1.17		0.051
	6.94	7.38	6.5	8.26	7.63	7.78	
Digit span backward		5.69 ± 1.39			6.72 ± 1.13		0.09
	5.63	5.88	5.56	6.67	6.48	7.00	
**Benton**
Correct drawings		6.02 ± 1.59			7.21 ± 1.21		0.049*
	5.19	6.31	6.56	6.52	7.56	7.56	
Number of mistakes		6.27 ± 2.97			3.76 ± 1.96		0.15
	7.25	6.19	5.38	4.78	3.30	3.22	
**CERAD**
Total immediate recall		20.23 ± 3.13			23.41 ± 2.14		0.001**
	19.94	19.06	21.69	23.19	22.96	24.07	
Delayed recall		6.48 ± 1.36			8.41 ± 1.08		0.00003**
	6.31	5.81	7.31	8.41	8.04	8.78	
Percent recognition		96.88 ± 3.49			99.38 ± 1.24		0.007*
	97.19	96.88	96.56	98.89	99.63	99.63	
Intrusions		0.46 ± 0.64			0.31 ± 0.43		0.75
	0.56	0.25	0.56	0.41	0.19	0.37	
Recall drawings		8.73 ± 1.89			10.59 ± 1.69		0.05
	7.88	8.25	10.06	9.85	10.48	11.44	
**General screening tests**
MMSE		28.91 ± 0.65			29.27 ± 0.59		0.32
	28.81	28.88	29.06	29.22	29.33	29.26	
SKT		2.17 ± 2.11			1.09 ± 1.19		0.074
	2.0	2.06	2.44	1.33	0.74	1.22	
TFDD		42.63 ± 2.12			45.84 ± 1.76		0.0001**
	42.44	42.44	43.00	45.48	45.22	46.82	


*^∗∗^*p* < 0.006 Bonferroni-corrected, ^∗^*p* < 0.05 uncorrected.*

### ROI GM Volumes

**FIGURE 1:**
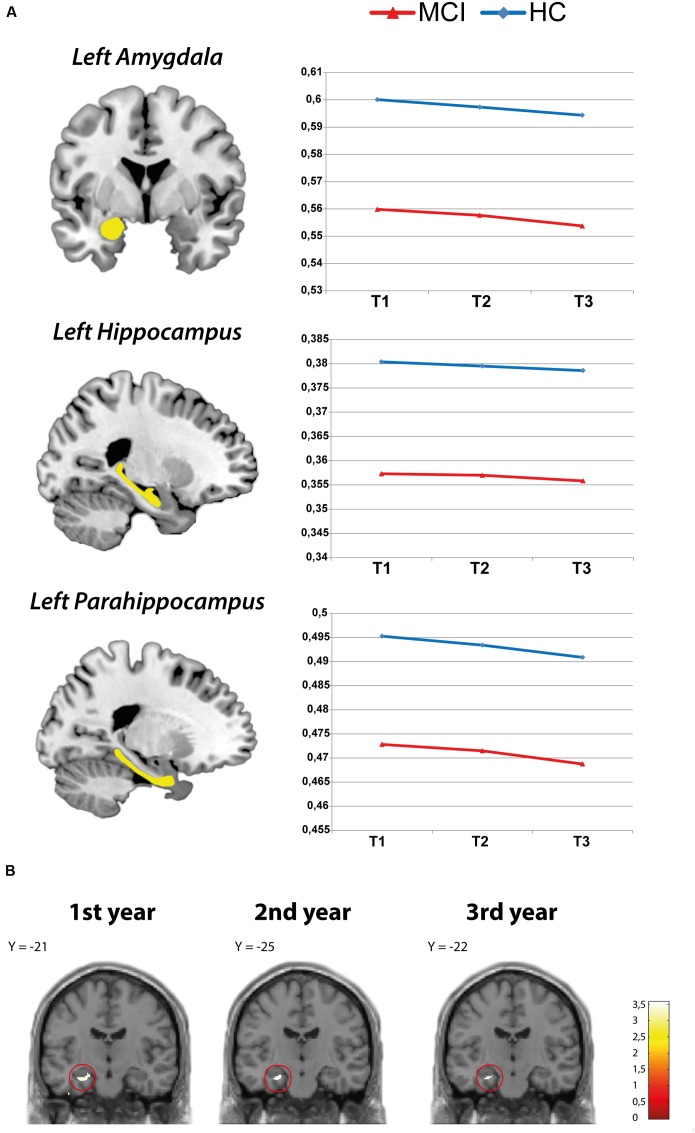
**(A)** Decline of region of interest (ROI) gray matter (GM) volume in the HC and mild cognitive impairment (MCI) group. **(B)** Whole-brain analysis: GM volume reduction in the amygdala and hippocampus (MCI > HC).

**FIGURE 2:**
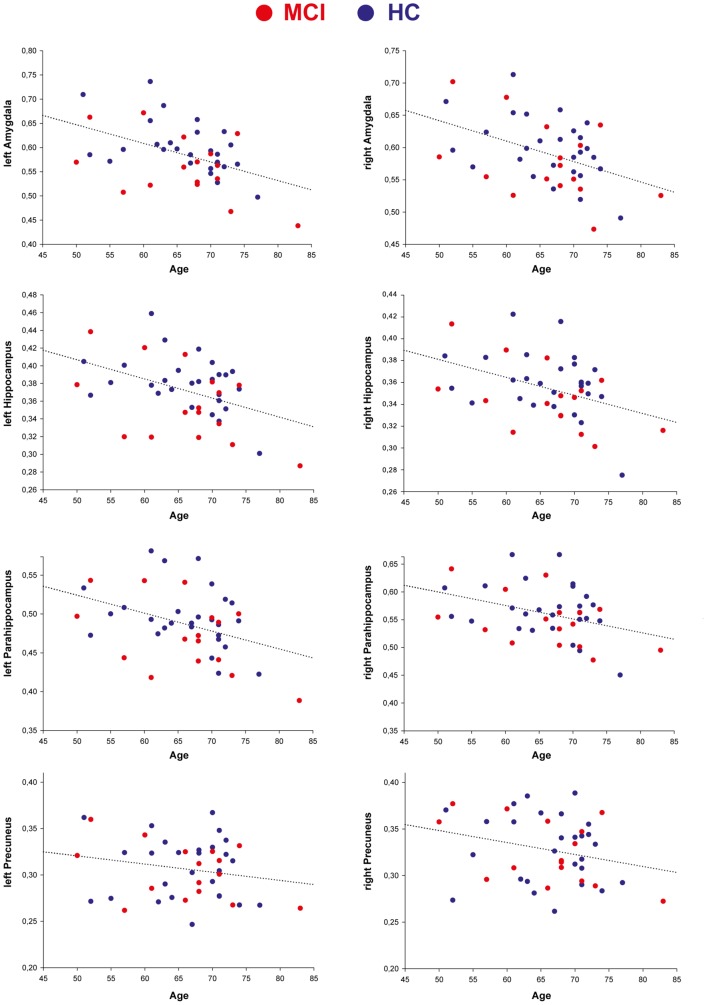
**Region of interest GM volume decrease across age in MCI and HC participants at T_1_**.

The LMM analyses on each ROI demonstrated a linear decrease in all ROI GM volumes in both groups over the course of the 3 years of observation. A group analysis between the MCI and HC group revealed significantly lower GM volume in the left amygdala, left hippocampus, and left parahippocampus in MCI participants compared to HCs at all three time points (see **Table 3**). Thus, MCI participants had significantly lower GM volume than controls in these areas already at the first time point, which then continued to decrease over the course of the following 2 years (effect of time in these ROIs: *p* < 0.001; see **Figure 1A**). However, there was no significant interaction between group and time point (*p* > 0.05), suggesting that atrophy rates were comparable between groups. Whole-brain analyses additionally indicated smaller left amygdala and left hippocampal volumes in the MCI compared to the HC group at each time point (see **Figure 1B**). No further activation outside of the ROIs was observed at *p* < 0.001. For visualization, **Figure 2** shows the decrease of ROI GM volumes across the age range of 50–83 years at T_1_ in MCI and HC participants.

**Table 3:** Results of the linear mixed model (LMM) analysis comparing mean region of interest (ROI) volumes between the MCI and HC group.

ROI		MCI			HC			MCI vs. HC
				
		T_1-3_ Mean ± SEM			T_1-3_ Mean ± SEM			T_1-3_ Mean
				
		T_1_	T_2_	T_3_	T_1_	T_2_	T_3_	*p*-value
Amygdala	Left		0.557 ± 0.01			0.597 ± 0.01		0.006^∗∗^
		0.559	0.557	0.553	0.600	0.597	0.594	
	Right		0.575 ± 0.01			0.595 ± 0.01		0.454
		0.578	0.575	0.572	0.598	0.595	0.592	
Hippocampus	Left		0.357 ± 0.01			0.379 ± 0.01		0.012^∗^
		0.357	0.356	0.355	0.380	0.380	0.379	
	Right		0.345 ± 0.01			0.359 ± 0.01		0.217
		0.346	0.345	0.344	0.359	0.359	0.358	
Parahippocampus	Left		0.471 ± 0.01			0.493 ± 0.01		0.048^∗^
		0.473	0.472	0.369	0.495	0.493	0.491	
	Right		0.546 ± 0.01			0.566 ± 0.01		0.342
		0.548	0.546	0.543	0.568	0.566	0.564	
Precuneus	Left		0.303 ± 0.01			0.307 ± 0.01		0.982
		0.304	0.303	0.302	0.307	0.306	0.305	
	Right		0.324 ± 0.01			0.328 ± 0.01		0.859
		0.325	0.325	0.323	0.329	0.328	0.327	


*^∗∗^*p* < 0.006 Bonferroni-corrected, ^∗^*p* < 0.05 uncorrected.*

### Associations between ROI GM Volumes and Neuropsychological Performance

Relating mean neuropsychological performance with mean ROI GM volumes across all 3 years within the whole sample showed that the association between CERAD total immediate recall performance and larger GM volume in the left amygdala (partial *r* = 0.49, *p* < 0.002), left hippocampus (partial *r* = 0.51, *p* < 0.001), and left parahippocampus (partial *r* = 0.52, *p* < 0.001) remained, indicating a robust relationship between CERAD total immediate recall performance and GM volume in these regions. In addition, WMS-R digit span forward performance was linked to larger left parahippocampal volume (partial *r* = 0.44, *p* < 0.006).

**FIGURE 3:**
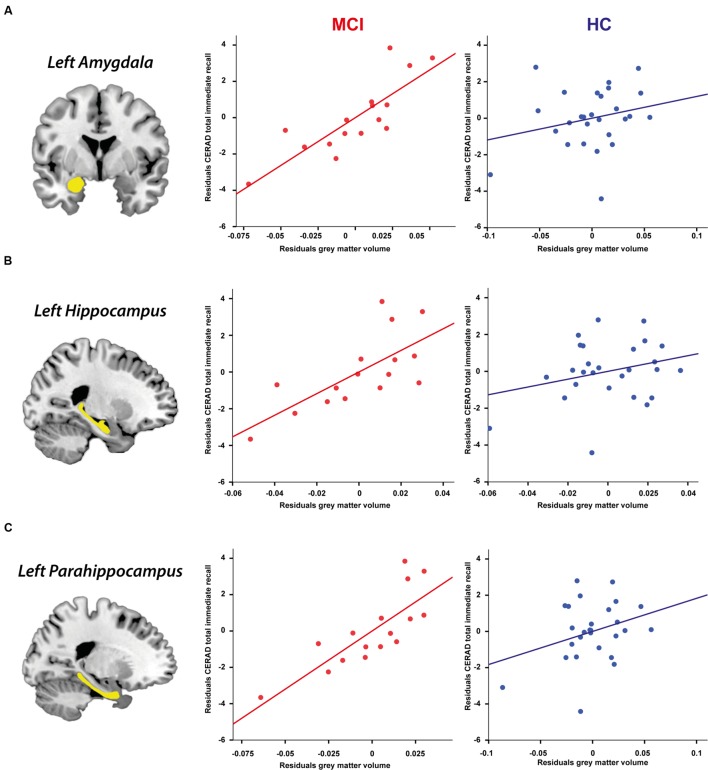
**Partial correlations controlling for age, gender, education, and TIV between CERAD total immediate recall performance and ROI GM volume in the MCI and HC group across 3 years (Holm–Bonferroni corrected *p* < 0.006).** **(A)** Left amygdala, **(B)** Left hippocampus, **(C)** Left parahippocampus.

Partial correlations between neuropsychological performance and ROI GM volumes within each group revealed that the abovementioned associations were solely driven by the MCI group as there were no significant correlations between neuropsychological performance and ROI GM volume in the HC group (all *p* > 0.05). In the MCI group, CERAD total immediate recall performance was highly correlated with GM volume in the left amygdala (partial *r* = 0.83, *p* < 0.001), left hippocampus (partial *r* = 0.72, *p* < 0.006), and left parahippocampus (partial *r* = 0.80, *p* < 0.002), implying a robust relationship between verbal memory impairment and GM volume reduction in these regions. MMSE scores showed no correlation with ROI GM volumes (all *p* > 0.05). **Figure 3** visualizes the relationship between mean CERAD total immediate recall performance and mean GM volumes of the left amygdala, hippocampus, and parahippocampus in the MCI and HC group across the three time points.

To find out to what extent GM atrophy in these ROIs significantly predicted verbal memory impairment in MCI participants already at the first measurement point, a stepwise linear regression was performed with the residuals (i.e., controlling for age, gender, education, and TIV) of CERAD total immediate recall performance at T_1_ as dependent variable and the residuals of GM volumes of the left amygdala, hippocampus, and parahippocampus at T_1_ as predictors. From this analysis, a significant model emerged [*F*(1,24) = 5.79, *p* < 0.05, *R*^2^ = 0.29], revealing that GM atrophy specifically in the left parahippocampus accounted for 29% of the variance in CERAD total immediate recall performance (β = 0.54, *p* < 0.05). Thus, left parahippocampal atrophy significantly predicted verbal memory impairment in the MCI group already at the first measurement point.

## Discussion

The aim of this longitudinal imaging study was to identify the relationship between neuropsychological and neuroanatomical changes associated with early stages of MCI identified by means of a thorough neuropsychological test battery in individuals unaware of cognitive impairment. Although several previous studies investigated the longitudinal trajectory of neuroanatomical alterations underlying MCI, these studies diagnosed MCI on the basis of a single general screening tests, lacked an analysis of these changes in relation to neuropsychological performance (e.g., Besson et al., 2015; Callahan et al., 2015; Dugger et al., 2015; Fellhauer et al., 2015; Granziera et al., 2015), or included subjective complaints as a diagnostic criterion. The present study aimed to overcome these limitations. Individuals classified as MCI who were unaware of beginning cognitive impairment exhibited verbal memory deficits (indicative of amnestic MCI) and GM atrophy in the left amygdala, hippocampus, and parahippocampus, compared to controls. Atrophy rates were comparable between groups, in contrast to our hypothesis of faster atrophy rates in MCI individuals than in controls. In line with our prediction of a specific relationship between GM atrophy and behavioral impairment in MCI individuals only, GM atrophy in the MCI group, but not the control group, was highly correlated with impaired verbal memory (CERAD total immediate recall). Moreover, GM atrophy in the left parahippocampus significantly predicted verbal memory impairment in MCI individuals already at T_1_, even without subjective awareness of cognitive impairment. This confirms our prediction of GM atrophy being evident in MCI individuals before they become aware of cognitive decline.

Lower GM volumes in the left amygdala, hippocampus, and parahippocampus are in accordance with previous studies reporting GM atrophy in the left MTL in MCI patients compared to controls (Ferreira et al., 2011; Nickl-Jockschat et al., 2012; Weiner et al., 2013; Csukly et al., 2016). Here, GM volume reduction in these regions was significantly linked to impaired total immediate recall performance (CERAD) in the MCI group, suggesting that GM atrophy in the MTL is specifically associated with verbal memory impairment. This finding complements previous observations that cerebral atrophy correlates with measures of general cognition in MCI (Jack et al., 2010). Atrophy rates did not significantly differ between MCI participants and controls, although this might be expected with progressing MCI (e.g., Trivedi et al., 2006; for a review, see Chetelat and Baron, 2003). However, this absence of differences in atrophy rates may be explained by the restricted time frame of the present study, comprising 3 years, which was probably not long enough to capture significant differences in atrophy rates. Moreover, none of the study participants converted to dementia during the course of the investigation, indicating that indeed early-stage MCI was captured here, thus further explaining the absence of group differences in GM atrophy rates. This could also explain why no GM atrophy in the precuneus was identified here, suggesting that atrophy in this region may occur at later stages of MCI. Note that MCI participants exhibited lower GM volumes compared to controls already at the first time point. This suggests that GM atrophy in the MCI group had already begun prior to enrolment in the study, warranting the inclusion of younger participants (<50 years) in future studies to be able to identify the point of divergence in medial temporal GM atrophy between MCI and normal aging.

According to the model of dynamic biomarkers of AD proposed by Jack et al. (2010), structural MRI is the last biomarker in the staging of the disease to become abnormal, preceded by Aβ-plaque biomarkers that are dynamic in early stages before the appearance of clinical symptoms, and by biomarkers of neurodegeneration that occur at later stages and correlate with symptom severity. MRI, FDG-PET, and CSF tau biomarkers are already abnormal in the MCI phase preceding AD. Findings of specific relationships between GM atrophy and neuropsychological impairment as observed in this study may be used in the future to increase prediction accuracy for conversion from MCI to AD.

The MMSE, a general cognitive screening test that has been commonly used to assess MCI in previous research did not show any correlations with GM volumes in the present MCI group and could not distinguish between the MCI and HC group: Both groups had an average score of 29 on the MMSE and MMSE scores remained intact over the course of the 3 years, with none of the participants having less than 27 points on the MMSE at any time point. Thus, the present MCI sample would not have been identified as such based on the results of this general screening test, despite evident cognitive impairment as demonstrated by significantly worse performance on the VLMT and CERAD (and tendencies toward impairment in the Benton and WMS-R). This is in line with previous findings showing that despite good sensitivity and specificity for diagnosing dementia, the commonly used cutoff scores of the MMSE do not show good accuracy for discrimination of MCI, misidentifying most of these subjects as having normal cognitive function (Kaufer et al., 2008). Since the MMSE is widely used in the MCI literature – and considering that individuals are classified as MCI with scores ranging from 23.1 to 28.7 (Nickl-Jockschat et al., 2012) – this not only entails the risk to include already more severely impaired individuals in MCI studies, but also causes problems for the comparison and evaluation of the results due to high variability in MCI diagnosis. Future studies should thus avoid relying on the MMSE alone and instead use a broader, more sensitive neuropsychological test battery to diagnose MCI and assess its severity. According to the present results, the total immediate and delayed recall scales of the VLMT and the CERAD seem especially sensitive to identifying early stage MCI in the absence of subjective awareness of cognitive decline. In contrast to the MMSE, the TFDD, a screening test aiming to detect early signs of dementia while differentiating cognitive problems due to depression, could distinguish between the MCI and the HC group in this study, although no between group differences on depression were detected. Thus, it may be worthwhile using the TFDD as an additional screening tool in future studies.

Importantly, SCCs are currently a diagnostic criterion for MCI. None of the individuals classified as MCI in this study were aware of any cognitive impairment (i.e., had no subjective complaints). Yet, the MCI group showed significantly worse performance on more than one test of the neuropsychological test battery. Confirming previous findings (Edmonds et al., 2014), this supports the idea to not include SCC as a diagnostic criterion for MCI in future studies in order to identify GM atrophy underlying early-stage MCI even without subjective awareness of beginning cognitive impairment.

### Limitations

The sample size of the present study was small due to a drop-out rate of almost 50%, resulting in only 43 participants (16 MCI) who completed all three measurement time points. High drop-out rates are an inherent risk in longitudinal studies, particularly those including elderly individuals. Future studies should thus aim to include more as well as younger (<50 years) participants in order to pinpoint at what age structural brain changes in relation to beginning cognitive impairment become evident and divergent from normal aging.

## Conclusion

The present findings indicate that GM atrophy in the left MTL underlying MCI is specifically associated with verbal memory impairment. This underlines the importance of combining neuroanatomical markers of MCI with specific neuropsychological tests as it implies that age-related GM atrophy is only predictive of MCI if accompanied by specific cognitive deficits. Moreover, our findings show that reductions in GM volume are evident even if individuals are not yet aware of cognitive impairment, demonstrating that individuals with neuroanatomical evidence of atrophy in regions associated with verbal memory impairments do not necessarily need to experience subjective cognitive concerns before meeting neuropsychological criteria for MCI. Further, general cognitive screening test such as the MMSE may not be sensitive enough to identify early-stage MCI. These findings have important clinical implications as they highlight the need to discard SCCs from MCI diagnosis. Moreover, it would be useful to apply comprehensive neuropsychological batteries, possibly by means of computerized cognitive assessments rather than relying on a single cognitive screening test to identify MCI. Lastly, establishing regular neuropsychological testing for MCI already in individuals below the age of 50 years will help identify MCI at early stages, enabling early intervention and a timely start of treatment.

## Author Contributions

Conception: UH. Organization: UH, KG, and MV. Execution: KG, MV, ED, and SE. Statistical Analysis: KG, MV, and GC. Manuscript: Writing of the first draft: KG and MV. Review and Critique: KG, MV, ED, SE, GC, and UH. All authors approved the final version of the manuscript.

## Conflict of Interest Statement

The authors declare that the research was conducted in the absence of any commercial or financial relationships that could be construed as a potential conflict of interest.

## Footnotes

1 http://marsbar.sourceforge.net/
